# Unusual Case of Extraction of Maxillary Lateral Incisors and Mandibular Central Incisors

**DOI:** 10.1155/2017/2486274

**Published:** 2017-05-23

**Authors:** Rosa-María Yañez-Vico, Maria Cadenas de Llano-Perula, Enrique Solano-Reina

**Affiliations:** Department of Stomatology, School of Dentistry, University of Seville, Seville, Spain

## Abstract

**Introduction:**

This article's purpose is to report a case where maxillary lateral incisors and mandibular central incisors are extracted and a canine substitution was performed as the best therapeutic option in order to obtain symmetry in a malocclusion with an upper lateral incisor with poor prognostic, solve moderate crowding, get enough space for the permanent dentition, and provide stability to the results.

**Case Report:**

An 11-year-old boy with straight profile with acute-to-normal nasolabial angle and protruded lips, mixed dentition, lower and upper severe crowding, and a bilateral molar angle Class I. The left maxillary lateral incisor failed endodontic treatment secondary to an intrusive traumatic lesion in the primary and permanent dentition. The treatment of choice was the extraction of both upper lateral incisors and both central lower incisors. The patient finished with molar and canine angle Class I and coincident midlines and was functionally stable; both lateral and protrusive jaw movements were effectively made by the first premolars and central incisors and canines without improper contacts of the rest of the teeth. Overbite of one-third and correct overjet were also achieved, and the esthetic outcome was satisfactory due to the composed material restorations of both the central and lateral incisors, as well as recontouring of the first maxillary premolars.

## 1. Introduction

It is widely accepted that, without extractions, it would be extremely difficult to solve some skeletal-dental discrepancies, and normal occlusion could be compromised if we induce severe protrusion in order to keep all the teeth in mouth [1]. The main objective of dental extraction for orthodontic purposes is to provide a level of aesthetics, functionality, and stability that could not be provided with any other method. First or second premolars have usually been the teeth of choice because of their proximity to the incisor area, which enabled their straightening and correction [2]. However, no preestablished patterns can be used for every single case, and careful diagnosis and planning must be made prior to any decision. On those cases where we consider following an unusual extraction pattern, periapical, panoramic, and occlusal X-rays, cephalograms, photographs, and models are required, and it is also essential to perform a diagnostic setup to be studied by a whole team of specialists.

Although uncommon, incisor extraction can be a viable treatment alternative in orthodontics [3]. Their extraction is often a treatment option in cases with anterior teeth discrepancy. The best circumstances for the extraction of mandibular incisors are Class I malocclusions with severe anterior discrepancy, with small lateral incisors or even agenesis of maxillary incisors and/or very wide mandibular incisors [4]; cases of lower severe crowding and correct posterior relationships; Class I malocclusions with anterior crossbite due to crowding and protrusion of the lower incisors [5]; tendency to Class III with relatively small crowding and incisors with a nontriangular form or anterior crossbite or incisors with edge-to-edge relationship, showing a tendency towards anterior open bite and malformed or periodontally compromised mandibular incisor [6].

When the maxillary lateral incisors are also extracted, treatment options are similar to the ones we have in tooth agenesis: space closure or space distribution with canine substitution [7]. To determine which treatment option would best suit our patient, different factors must be taken into account, such as the skeletal pattern, the amount and direction of remaining growth, the profile, the type of malocclusion our patient has, the amount of overjet, and the total number of extractions we are going to perform. We must also consider the size, colour, and shape of the maxillary canines, their gingival margin level, and the smiling lip level [8].

Space closure has been reported to be [9] a viable procedure, functionally stable, and with satisfactory aesthetics due to the late improvements in contouring and reshape of restorative treatments and the use of new materials such as hybrid composite resin. On the other hand, we are unable to accurately predict the survival rate of an implant in case of choosing space reopening as a treatment.

This article's purpose is to report a case where maxillary lateral incisors and mandibular central incisors are extracted and a canine substitution was performed as the best therapeutic option in order to obtain symmetry in a malocclusion with an upper lateral incisor with poor prognostic, solve moderate crowding, get enough space for the permanent dentition, and provide stability to the results.

## 2. Diagnosis and Etiology

**Figure 1:**
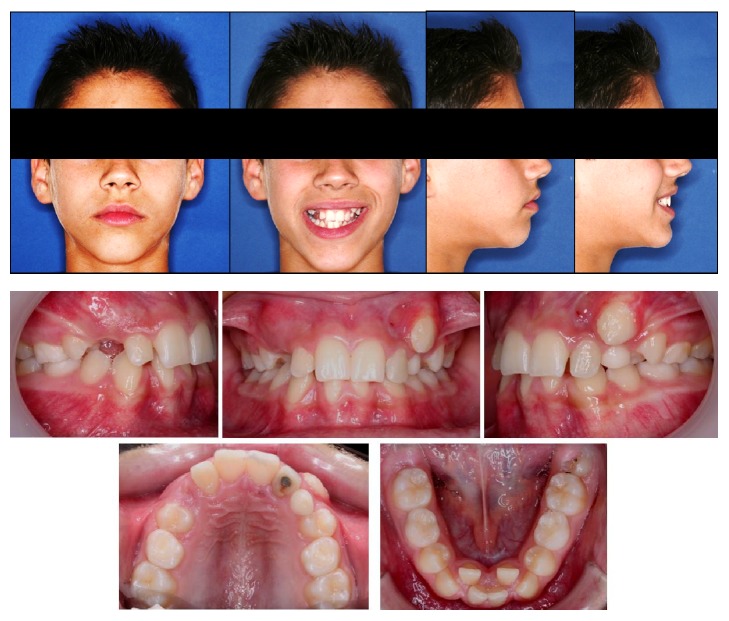
Initial diagnostic photographs.

**Figure 2:**
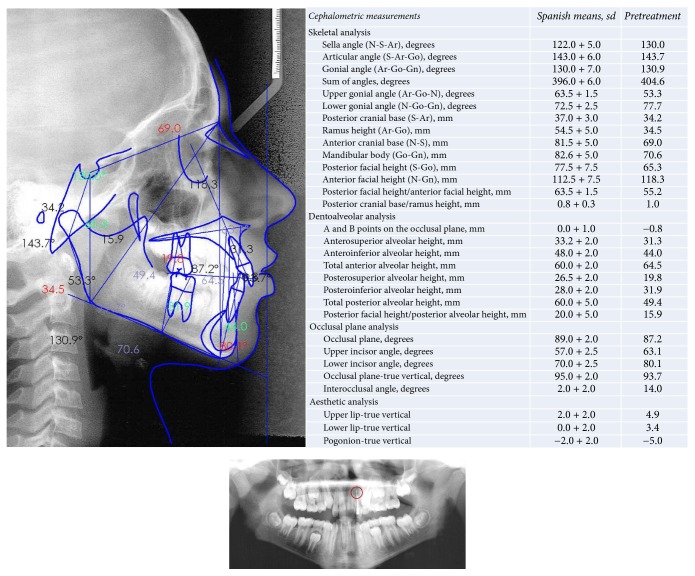
Panoramic radiograph, lateral radiographic records, and cephalometric measurements.

An 11-year-old boy came to the Orthodontics Department of our Dentistry School (University of Seville, Spain) with the chief complaint of “incorrectly placed teeth.” He had a straight profile, mixed dentition, and a bilateral molar angle Class I. The canine relationship was still uncompleted due to eruption. The midlines were not coincident (the lower midline was 1 mm more to the right than the upper one) and he had a 1.5 mm overjet, two-thirds overbite, and moderate crowding (arch length discrepancies: maxilla, −5 mm; mandible, −6 mm). No signs of temporomandibular problems were found (Figure 1). The left maxillary lateral incisor was darker than the other incisors, and a failed endodontic treatment of that tooth was shown on the panoramic rx, secondary to an intrusive traumatic lesion in the primary maxillary lateral incisor and also in the permanent maxillary lateral incisor [10, 11] (Figure 2).

The cephalometric analysis showed that the patient was dolichofacial (mandibular plane angle 33.7°, gonial angle 130.9°), with moderate skeletal Class II (convexity 5.9 mm, Wits appraisal: −0.8), and had a Class I occlusal plane angle (87.2°) and a lower facial height of 45.5°. Tooth axial inclination was 63.1° for the maxillary incisor and 80.1° for the mandibular incisor (both lingually inclined) and an open interincisal angle (143.2°) (Figure 2).

## 3. Treatment Objectives

The treatment objectives were to

- maintain molar angle Class I and obtain canine Class I without affecting the profile;
- achieve coincident midlines, solving crowding and avoiding incisor flaring;
- prepare the arches for the proper eruption of the permanent teeth;
- avoid upper and lower arch constriction due to unusual incisors extraction pattern;
- obtain adequate final smile esthetics.

## 4. Treatment Alternatives

The treatment of choice was the extraction of both upper lateral incisors and both central lower incisors. We decided upon such an unusual extraction pattern because according to the cephalometric analysis and the data gathered from the casts the extraction of incisors would help us solve the crowding in a very stable way without harming the esthetics in the anterior front, the canines would substitute the laterals, and the first premolars will work as canines. The fact that the left maxillary lateral incisor had to be removed due to a traumatic lesion and failed endodontic treatment and initial signs of ankylosis helped us decide in favor of this choice.

The usual extraction pattern (four bicuspids) would have resulted in a more retrusive profile, and we will still be in need of an implant or other prosthetic solution for the lateral incisor that had to be extracted. Extraction of both first maxillary bicuspids together with stripping in the lower arch would have also been possible, but the mandibular incisors had a small size and the results could have been more prone to relapse.

## 5. Treatment Progress

The surgical extraction of the left maxillary lateral incisor was performed as the first step for our treatment, so that the canine could erupt in its place. Then, the other maxillary lateral was extracted as well as both mandibular central incisors.

**Figure 3:**
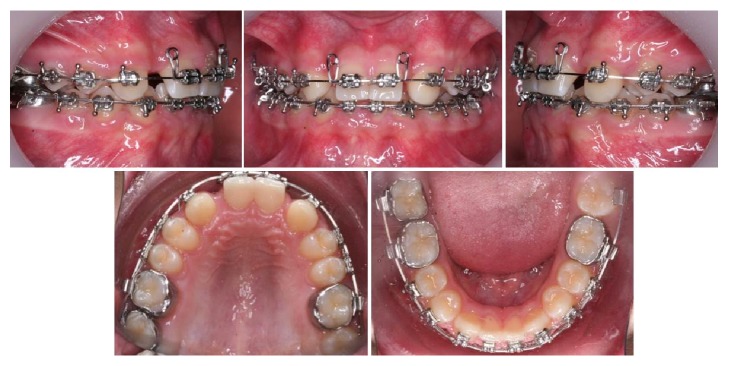
Customized 0.016 × 0.022′′ stainless steel archwire with two loops distal to the central incisors.

Multibracket fixed appliances were placed avoiding the remaining temporary teeth and a NiTi round 0.014′′ archwire was used for the early phases of alignment. Once proper leveling was achieved, we placed a 0.017 × 0.025′′ upper NiTi archwire and started grinding the canines' cuspid to avoid interferences in occlusion, as they were going to be used as laterals. A utility arch was placed on the upper arch in order to intrude the incisors, constructed in 0.016 × 0.022′′ stainless steel, and once we had the desired position of the central incisors, the space between them and the canines was slowly closed using a customized 0.016 × 0.022′′ stainless steel archwire with two loops distal to the central incisors (Figure 3). The upper canines were overcorrected in order to imitate the natural position of lateral incisors.

## 6. Treatment Results

**Figure 4:**
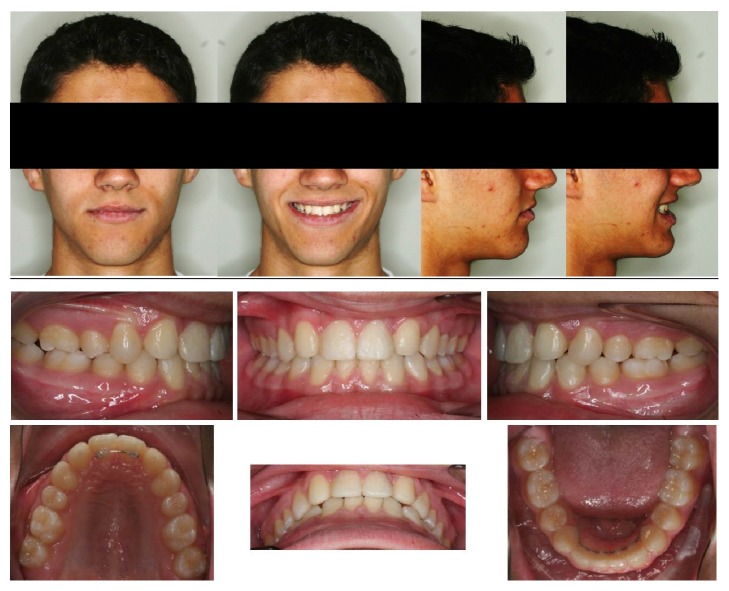
Final diagnostic photographs.

The patient finished with molar and canine angle Class I and coincident midlines and was functionally stable; both lateral and protrusive jaw movements were effectively made by the first premolars (now working as canines) and central incisors and canines (now working as lateral incisors) without improper contacts of the rest of the teeth. Overbite of one-third and correct overjet were also achieved, and the esthetic outcome was satisfactory due to the composed material restorations of both the central and lateral incisors, as well as recontouring of the first maxillary premolars. Gingivectomy was also needed in order to get suitable gingival levels for every anterior upper tooth, and fixed retention was placed in both the upper (1+1) and the lower arch (3-3) in order to minimize the chances of relapse (Figure 4).

**Figure 5:**
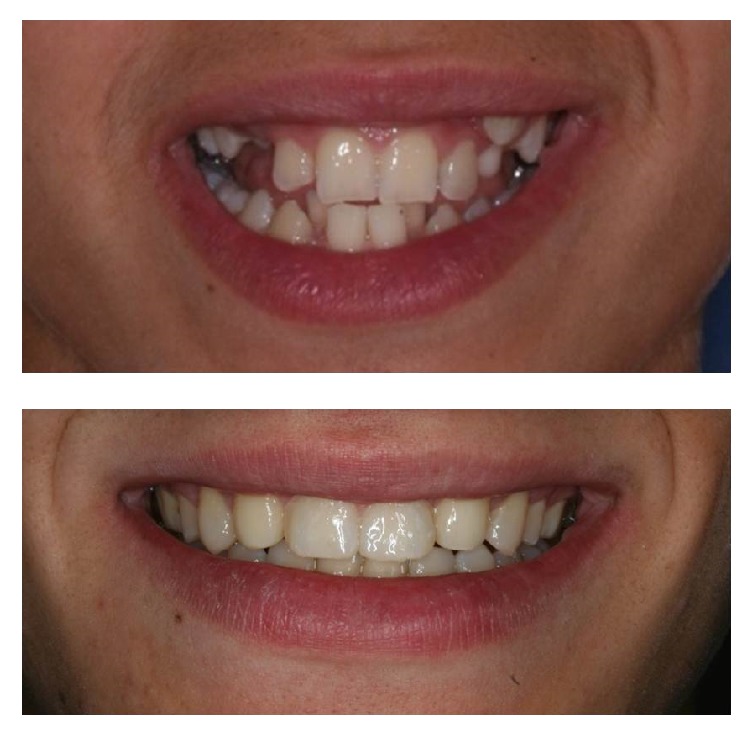
Initial and final smile photographic records.

**Figure 6:**
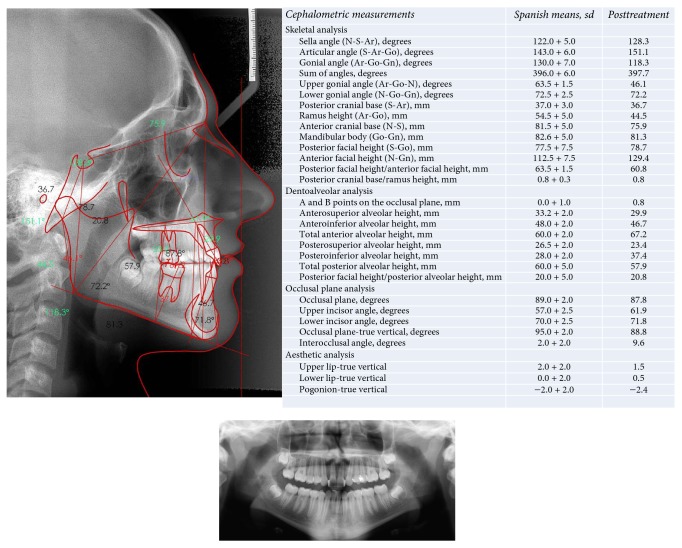
Final panoramic radiograph and lateral radiographic and cephalometric measurements.

**Figure 7:**
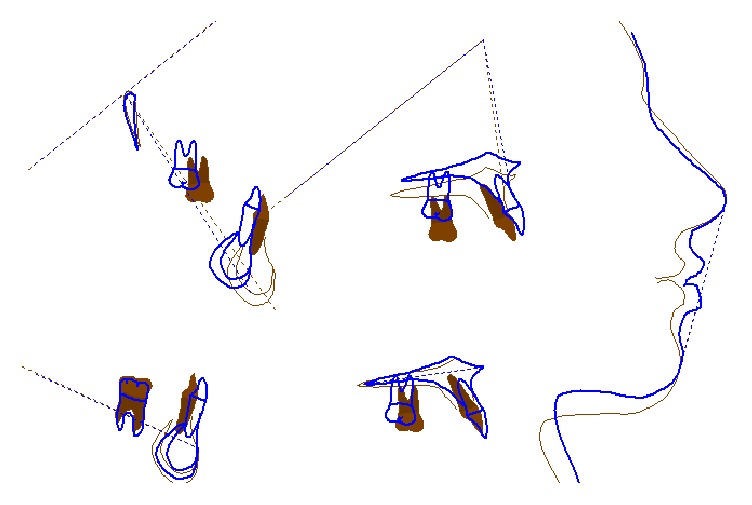
Superimpositions (blue: pretreatment; red: posttreatment).

The smile is more symmetric and greatly improved and the incisal smile curve is now parallel to the inner contour of the lower lip (Figure 5). Patient shows a reasonable good profile with anterior teeth providing support for the upper and lower lips. The convexity varied from 5.9 mm (Class II) to 1 mm (Class I), because of to the improved position of the incisors; tooth axial inclination was buccally straightened 1.2° for the upper incisor and 8.3° for the lower incisor, and the interincisal angle was decreased to normality (from 143.2° to 133.6°) (Figures 6 and 7). Nevertheless, gingival margins are not ideal; 1.3 is not well leveled on 1.2.

## 7. Discussion

In this case report, there are two key aspects, the choice of extracting incisors, both maxillary and mandibular, and the canine substitution. Although orthodontic patients with missing maxillary laterals are often seen in our offices [12], in fact after wisdom teeth the lateral maxillary incisor and the second mandibular premolar are the most common missing teeth [13]; it is extremely rare to decide their extraction as a therapeutic option, with exception of pathological conditions that might compromise their viability such as severe root resorption or pulpal/periodontal infection [14]. Classically, space reopening and prosthetic substitution of the laterals had been the most accepted treatments. Some of the disadvantages of space closing reported in literature are undesired space loss, possibility of space reopening and relapse [15], need for long term fixed retention and restoration of the upper front [16], destruction of inclined plane relationship, and inadequacy of canine occlusal adaptation as a lateral incisor due to its form and size. Henns in 1974 [17] proved however that the permanent changes in the arch when canines were used as laterals were less than 1,5 mm, and, since then, space closure has turning into a preferable treatment both periodontally and occlusally. Zachrisson and Mjör in 1975 [18] also made a histological analysis of grinding during the recontouring of the canines, reaching the conclusion that it was harmless to pulpal tissues when performed on young teeth and with sufficient irrigation. Nordquist and McNeill [19] found that patients with maxillary lateral incisor spaces closed were healthier periodontally than the ones who received prosthetic laterals and found no differences regarding the occlusal function between those two groups. Later, Robertsson and Mohlin [6] sustained those findings and added that patients without prosthetic laterals were happier with the result and that no signs of temporomandibular distress were found in any of them. It is also a preferred treatment because of the stability of the finished results and the possibility of completing the treatment in the adolescence, so that alveolar bone is maintained because of the early translation of the canines to the site of the laterals [9, 20].

In order to decide what option would best suit our patient there are some criteria that we must attend: firstly, the type of malocclusion [21]; secondly, the posterior occlusal relationship; thirdly, the amount of crowding or existence of diastemas; fourthly, the need for extractions or the arch length deficiency; fifthly, the pretreatment position of the canines; and, lastly, the amount of alveolar protrusion. It is widely accepted [8, 9, 22] that space closure can be the best option when facing molar and premolar Class II or Class I with space deficiency and that, on the other hand, we must decide upon space reopening in Class III or Class I with extra space. Secondly, the width, shape, colour of the crown, and length of the roots of the adjacent canines must be also taken into account for a more successful case ending, as well as the wideness and height of the smile and gingival margin of the canine, which should resemble the one on the lateral. Although this can be considered more a prosthetic than an orthodontic work, after space closure, recontouring by grinding of the canines, composite restoration, or gingivectomy might be also needed. Brough et al. [23] showed in their 2010 paper that the morphology, size, and shape of the canines can have an intense effect on perceived smile attractiveness among general population, dentists, and orthodontists.

Regarding the extraction of lower central incisors, although, according to Proffit, their extraction accomplished nearly 20% of the extraction cases before 1950, it has truly become a rare choice nowadays. Detractors argue that increased overbite and overjet can be produced, as well as space reopening, unaesthetic loss of interdental papillae in the mandibular anterior region [24], relapse, mesial tipping of canines, lingual tipping of the remaining incisors, inadequate creation of space to correct crowding, sometimes even an increase in the maxillary incisor crowding, and a lack of concordance of the maxillary and mandibular midlines, when performed unilaterally [25].

According to the literature, the indications of the extraction of mandibular incisors are moderate Class III malocclusions, tendencies with an edge-to-edge occlusion of the incisors, anterior crossbite and minimal overbite or open bite tendency [26], relative mandibular tooth size excess, mandibular tooth size-arch length discrepancy [27], structurally or periodontally compromised teeth, supernumerary mandibular incisors, ectopic eruption of mandibular incisors [28], and temporomandibular joint dysfunction cases with retroposition of the mandible [25], along with other asymmetric conditions [28–30]. On the other hand, contraindications of this kind of extractions have been described as significant anterior maxillary tooth size excess [31], deep overbite, periodontal disease, triangular shaped mandibular incisors, and increased overjet [32].

It should be mentioned that this type of treatment, despite the fact that it might compromise initial smile esthetics due to anterior extractions, allows total space closing and stable occlusion results in the young age as it is the case of the present patient. From a psychological point of view, preparing a clinical case for long term space maintaining for future implant or prosthetic rehabilitation often is not a preferable option due lack of immediacy in the complete therapeutical solution and this might lead to some type of patient's [33] unsatisfaction [34].

It is also important to point out that there is a lack of prospective studies or clinical trials in literature regarding the benefits of the extraction of incisors for orthodontic purposes, as assessed by Zhylich and Suri's [35] systematic review of 2011, so we have to behave according to the case reports or case series published, with no real evidence of the advantages and disadvantages of such procedures.

In this case report, moderate crowding of the lower arch was present (mandibular discrepancy −6 mm) with a lack of space also in the upper arch (Maxillary discrepancy −5 mm) and increased size of the maxillary central incisors and canines, which were still erupting. Molar angle Class I was present in both left and right sides and a slight asymmetry of the midline was observed. After the extraction of both maxillary lateral incisors and central mandibular incisors, canine angle Class I was achieved and molar angle Class I was maintained, with appropriate functional guides. Midlines were also coincident and upper canines were used as laterals performing grinding and prosthetic reconstruction. First premolars were transformed as canines. Fixed retainers were placed in both upper and lower arches as advised in cases of space closure. Retention and adequate functional guides should warrant the case to be stable, and there is a proper aesthetic result of the smile, with harmonic gingival levels of both laterals and canines.

When it comes to the functional aspects of this case, it is important to know that first maxillary premolars are going to be placed instead of the canines, and because of their shorter roots they might not deal with occlusal forces as easily as canines do [36]. That is why erasing any interference of their lingual cusps by applying sufficient buccal root torch has been proposed in literature, producing at the same time a sort of canine eminence that will improve aesthetics. On first maxillary molars, we should also apply lingual crown torch to avoid having problems with their lingual cusps [8].

Retention is very important in cases of space closure. There are some measures that could help us get a more stable case, for example, overcorrection of tooth movement, ensuring parallel roots, careful fit of removable retainers, and final placement of fixed retainers. It has as well been advised that after a period of retention and settling the occlusion must be rechecked, to avoid further trauma [21]. Nevertheless, this type of unusual extraction pattern in orthodontics often requires long term fixed retention until any other type of relapse prevention methods would be clinically available [37].

This is a case that needs careful study and whose development involved many different specialists such as orthodontists, periodontists, and prosthodontist; thus, close work with other professionals is key for a successful outcome; aesthetic results should be as similar as possible to natural dentition and be life-long lasting.

## 8. Summary and Conclusions

Although uncommon, therapeutic incisor extraction and canine substitution can be a viable treatment when traumatic/septic conditions might affect anterior teeth viability and there is moderate to severe crowding and correct posterior relationships. Cases must be carefully selected and they usually involve a whole team of specialists from the first to the last phases of treatment, as gingivectomy, recontouring, and reshape of the canines, premolars, and incisors are usually needed.
